# Comparison of the First and Second Wave of Infections by SARS-CoV-2: A Retrospective and Longitudinal Study From a Primary Health Care Center in Santiago of Chile

**DOI:** 10.3389/fpubh.2022.913519

**Published:** 2022-06-30

**Authors:** Claudio Acuña-Castillo, Ailen Inostroza-Molina, Sergio A. Castro, Sonia Molina-Cabrera, Elías Leiva-Salcedo, Denise Riquelme, Roberto Luraschi, Carlos Barrera-Avalos, Eva Vallejos-Vidal, Andrea Mella-Torres, Daniel Valdés, Claudio Torres, Kevin Maisey, Alejandro Escobar, Sebastián Reyes-Cerpa, Daniela Toro-Ascuy, Mónica Imarai, Felipe E. Reyes-López, Ana María Sandino

**Affiliations:** ^1^Centro de Biotecnología Acuícola, Facultad de Química y Biología, Universidad de Santiago de Chile, Santiago, Chile; ^2^Departamento de Biología, Facultad de Química y Biología, Universidad de Santiago de Chile, Santiago, Chile; ^3^Centro de Salud Familia San José de Chuchunco, Santiago, Chile; ^4^Laboratorio de Ecología y Biodiversidad, Facultad de Química y Biología, Universidad de Santiago de Chile, Santiago, Chile; ^5^Centro de Nanociencia y Nanotecnología CEDENNA, Universidad de Santiago de Chile, Santiago, Chile; ^6^Facultad de Medicina Veterinaria y Agronomía, Universidad de Las Américas, Santiago, Chile; ^7^Department of Neurobiology Drexel University, Philadelphia, PA, United States; ^8^Laboratorio Biología Celular y Molecular, Facultad de Odontología, Instituto de Investigación en Ciencias Odontológicas, Universidad de Chile, Santiago, Chile; ^9^Centro de Genómica y Bioinformática, Facultad de Ciencias, Universidad Mayor, Santiago, Chile; ^10^Escuela de Biotecnología, Facultad de Ciencias, Universidad Mayor, Santiago, Chile; ^11^Laboratorio de Virología, Facultad de Ciencias de la Salud, Instituto de Ciencias Biomedicas, Universidad Autónoma de Chile, Santiago, Chile; ^12^Department of Cell Biology, Physiology, and Immunology, Universitat Autònoma de Barcelona, Barcelona, Spain

**Keywords:** SARS-CoV-2, COVID-19, waves of infection, vaccination, Chile

## Abstract

The current COVID-19 pandemic is caused by the severe acute respiratory syndrome coronavirus 2 (SARS-CoV-2). Many countries have reported the experience of at least two contagion waves, describing associated mortality rates and population behavior. The analysis of the effect of this pandemic in different localities can provide valuable information on the key factors to consider in the face of future massive infectious diseases. This work describes the first retrospective and comparative study about behavior during the first and second waves of the COVID-19 pandemic in Chile from a primary Healthcare Center. From 19,313 real-time quantitative PCR (RT-qPCR) tests assessed, the selected 1,694 positive diagnostics showed a decrease in mortality rate in the second wave (0.6%) compared with the first (4.6%). In addition, we observed that infections in the second wave were mainly in young patients with reduced comorbidities. The population with a complete vaccination schedule shows a decrease in the duration of symptoms related to the disease, and patients with more comorbidities tend to develop severe illness. This report provides evidence to partially understand the behavior and critical factors in the severity of the COVID-19 pandemic in the population of Santiago of Chile.

## Introduction

The COVID-19 pandemic collapsed the global public health systems at the beginning (1–3), due to the lack of information about it, both in its behavior and treatment. To date, most countries have experienced several waves of infections since the beginning of 2020 in Wuhan, China (4). The empirical data collected to date show differences in viral infection behavior between the periods in the population diagnosed with COVID-19, in age range, symptoms, and disease severity, in different countries and locations (5, 6). For example, patients in the second wave were younger, and the length of hospitalization and the case fatality rate were lower than those in the first wave in Spain, with differences in risk factors for mortality, such as comorbidities (7). On the other hand, Seong et al. (8) reported a higher case fatality rate in the third wave compared with the second (0.91 vs. 1.96%) in South Korea (8). While in Thailand, the infections and spread were enormously increased in the third wave, with significant differences between age ranges (5), possibly due to the lack of public and social health policies. In addition, the vaccination plan has decreased the rate of deaths and infections in different waves of infections (9). However, despite these data being handy for understanding the behavior of this and other possible massive infections in different countries and geographic regions, there is no report on the behavior of the SARS-CoV-2 virus in the first wave of contagion in Santiago of Chile. In this study, we analyzed the population's behavior from Centro de Salud Familiar San José de Chuchunco, a primary healthcare center in the Metropolitan region. We compared in SARS-CoV-2 positive-diagnosed patients the age, symptomatology duration, and severity between the first and second waves of SARS-CoV-2 infection and evaluated the effects of vaccination on the course of the second wave. Our analysis indicates that the first wave of infections occurred in the older population with a 4.5% mortality rate with a series of related comorbidities. This mortality rate decreased to 0.6% in the second wave, which is related to a younger population with fewer comorbidities and the beginning of mass vaccination of older patients. These results shed light and suggest the behavior of the COVID-19 disease at the beginning of the pandemic in Chile, one of the 15 best countries to live in times of pandemic according to The Covid Resilience Ranking (10).

## Materials and Methods

### Data From San José de Chuchunco Family Healthcare Center (Santiago of Chile)

We used the database of San José de Chuchunco, a primary healthcare attention center in the Metropolitan region of Santiago, Chile, to conduct a retrospective study for the first and second waves of infection of SARS-CoV-2 in a population of Chile between March 2020 and June 2021. A total of 19,313 real-time quantitative PCR (RT-qPCR) tests were performed, with 12.7% overall positivity (2,470). The first wave corresponds to the date between March and July 2020 (1,721 tests and 742 positives), the inter-wave between August 2020 and January 2021 (10,445 tests and 564 positives), and the second wave from February to June 2021 (7,147 tests and 1,164 positives). The beginning and end of the waves of infection are related to the sustained and significant increase and decrease in infections at the national level. The periods of the first and second waves of our study coincide with those officially reported for the country by the Ministry of Health of the Government of Chile (11). We obtained access to 1,694 exams used for this study. The analysis includes the first wave (666 positives), inter-wave (323 positives), and second-wave (705 positives) analysis, related to gender, symptoms prevalence, viral load, comorbidities, and effect of mass vaccination on health personnel and older adults (≥ 60-year-old).

Based on the patient's information, we calculated the severity of the infection, which was the summary of the values recorded in symptoms, hospitalization, and intensive care unit (ICU)/intensive treatment unit (ITU). This variable moves between 0 (patient with COVID-19 symptoms) and 3 (patient in ICU/ITU). To analyze the comorbidities, we quantified the number of comorbidities of each patient; this variable moves between 0 (no-comorbidity) and 17 (all comorbidity).

### Viral Load

To estimate quantitatively the viral load from patient samples, we constructed a standard curve of RT-qPCR by making serial 1/10 dilutions using the positive control TaqMan 2019-nCoV Control Kit v1 (104 copies/μl) (Thermo Fisher Scientific, Cat. No. A47533). By plotting the log10 copy number vs. Cq, the following equation of the line was obtained (y = −3.07 × X + 40.2). The Cq obtained from sample data in RT-qPCR was replaced in X.

### Data Representation and Statistical Analysis

Our analysis focused on the difference between the pandemic stage and post-pandemic stage in terms of record distribution. The dichotomic variables (gender, presence/absence of symptoms, death, or survival) were analyzed using chi-square and contingency tables, evaluating the homogeneity of these associations. Between March 2020 and June 2021, the continuous distribution of the variables and the number of symptoms per patient in each period were analyzed using one-way ANOVA. Then, we analyzed the weight of each factor in the determination of the fourth response variable: (a) vaccination (vaccinated vs. unvaccinated), (b) symptoms duration, (c) viral load, (d) severity of the illness, and (e) mortality. These analyses were performed using analysis of covariance (ANCOVA), where the age of the patients was used as a covariable, and the rest of the variables (pandemic stage, gender, presence/absence of symptoms, and vaccination) were factors. A *p*-value of < 0.05 was considered statistically significant. GraphPad Prism 8 statistical software was used to analyze and plot the data obtained.

### Ethics Statement

The data included in this study were authorized by the Ethical Committee of the University of Santiago of Chile (No. 226/2021) and the Scientific Ethical Committee of the Central Metropolitan Health Service, Ministry of Health, Government of Chile (No. 370/2021), and following the Chilean law in force.

## Results

**Figure 1:**
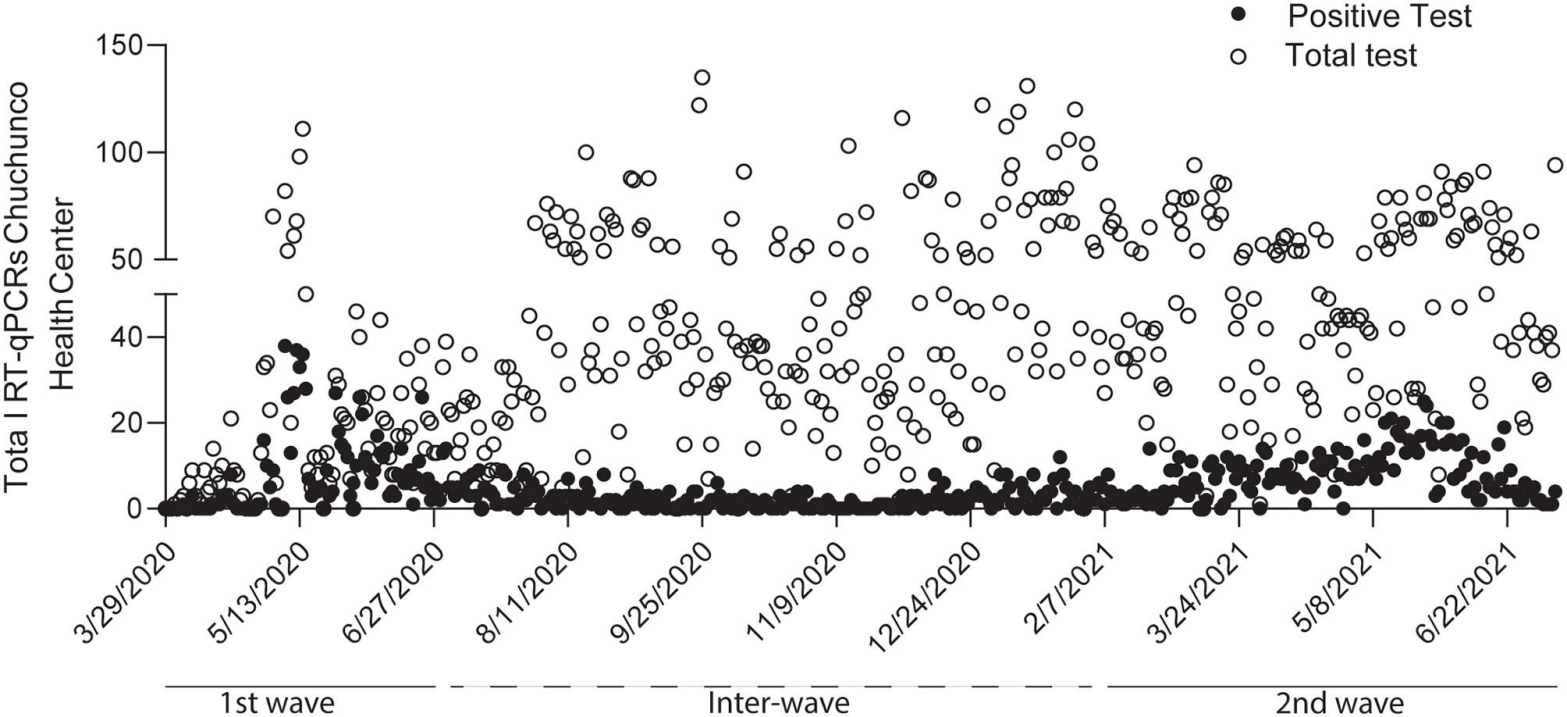
Confirmed patients in the Chuchunco primary health center during the first and second waves of infections in Santiago de Chile. The analyses include 1,694 patients distributed between the first wave (March to July 2020), inter wave (August 2020 to January 2021), and second wave (January to June 2021). The analyses include 666, 323, and 705 confirmed positive cases, respectively. Black circles correspond to positive cases, and open circles correspond to total tests carried out in the health center.

**Figure 2:**
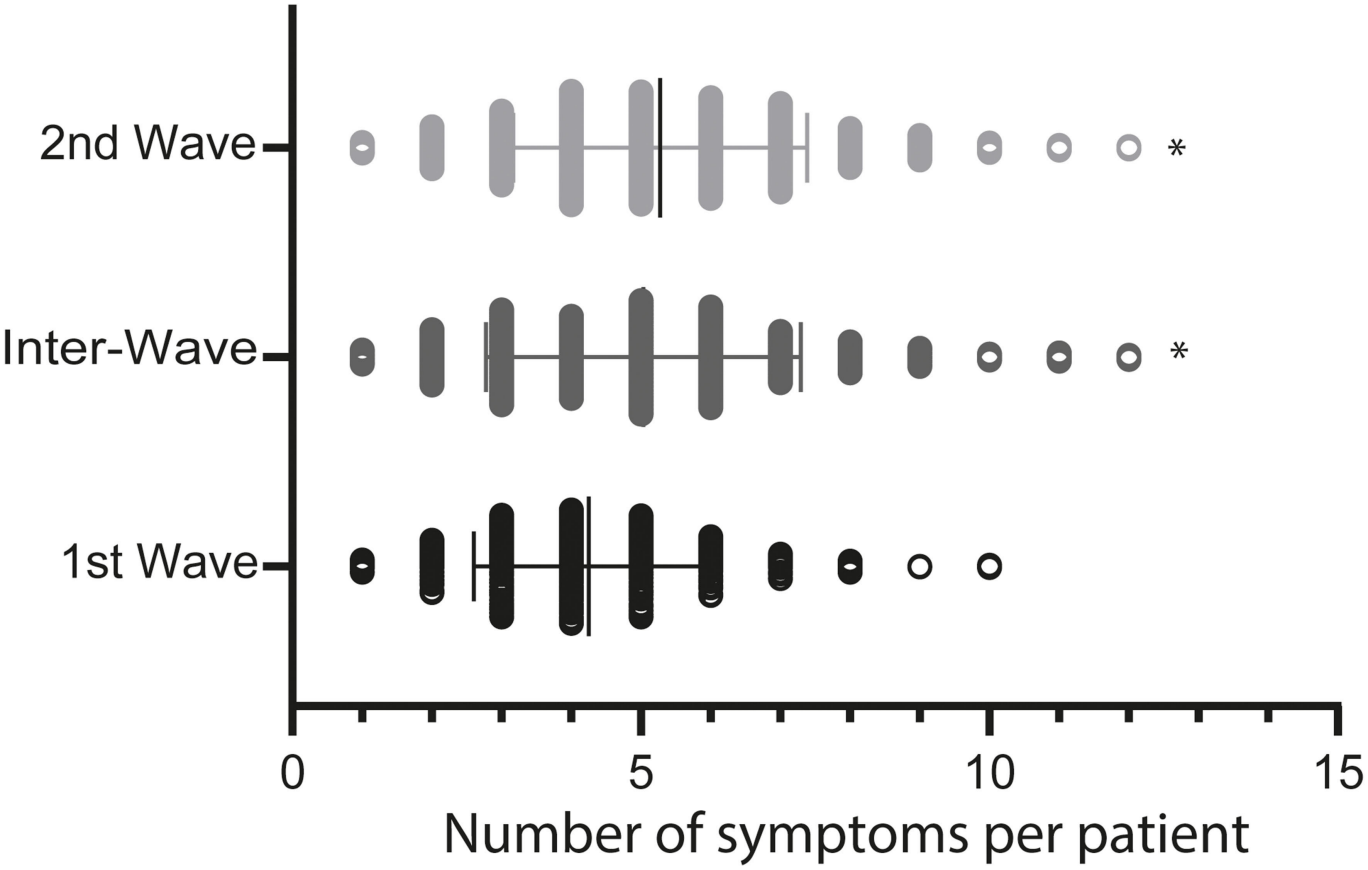
The number of symptoms per patient in the first, inter, and second waves. The graph corresponds to the number of symptoms per patient from the 1,694 positive tests per analyzed period. *p < 0.05. Each period is shown with the mean value.

The study included 1,694 positive patients; 666 corresponding to the first wave, 323 for inter-waves, and 705 in the second wave. The behavior of the first and second waves are shown in Figure 1. The black circles correspond to the positive cases, and the open circles to the total RT-qPCR performed on each day. The first wave of infections is shown for dates between March 2020 and July 2020, and the second wave is between February 2021 and June 2021. From the 1,694 diagnostics analyzed, 791 were men and 903 were women. The proportion of male and female patients differs from the expected proportion of 50% (*X*^2^ = 7.4; *p* < 0.05), implying that women make up a more significant proportion than men in the patient population in each wave. This difference remains unchanged in the three stages of the pandemic studied (Table 1; *X*^2^ = 2.1; *p* > 0.05). The mean age of patients changed depending on the pandemic stage; in the first wave, the mean age was 47.5 ± 17 years, between waves, it was 44.9 ± 17 years, and in the second wave it was 42.7 ± 16 years (Table 1), indicating a shift of the infected patients toward the younger population. In addition, we saw a change in the asymptomatic patients, 8% during the first wave, 9.3% between waves, and 5.2% in the second wave, with a greater number of symptomatic patients for the second wave of infections (Table 1). Moreover, the patient from the first wave presenting symptoms of longer duration (first wave = 17.5 ± 13 days; inter-wave = 11.6 ± 12 days; second wave = 11.5 ± 7 days) (Table 1, *F* = 74.2; *p* < 0.05). In the first wave, the main symptoms were cough (16%), headache (14%), myalgia (15%), and fever (10%). While in the second wave, an increase in respiratory distress (13%), gastrointestinal disease (14%), anosmia (13%), and headache (19%) were observed, with symptoms, such as asthenia and myalgia decreasing (Table 2). In addition, we observe that the number of symptoms per patient increases in the inter- and second wave compared with the first wave of infections (Figure 2).

**Table 1:** Clinical and epidemiological characteristics of patients with COVID-19 infection from Chuchunco health center.

**Parameters analyzed**		**1^**st**^ wave**	**Inter-wave**	**2^**nd**^ wave**	**Docima**
Patients (number)	Total	666	323	705	
Gender (number)	Women	355	183	365	
	Men	311	140	340	X^2^ = 2.1; *P* > 0.05
	Average Age	47.5 ± 17	44.9 ± 17	42.7 ± 16	*F* = 13.4; *P* <0.05
Symptomatology (%)	Asymptomatic	8.0	9.3	5.2	
	Symptomatic	92.0	90.7	94.8	X^2^ = 6.7; *P* <0.05
Survival rate (%)	Recovery	95.5	98.5	99.4	
	Mortality	4.5	1.5	0.6	X^2^ = 24.6; *P* <0.05
	Duration of symptoms (days; mean ± SD)	17.5 ± 13	11.6 ± 12	11.5 ± 7	*F* = 74.2; *P* <0.05
	Viral load (copies/μl)	1,15 x 107 ± 5,37 x 107	1,73 x 106 ± 7,0 x 106	4,56 x 105 ± 2,64 x 106	*F* = 236.8; *P* <0.05
Comorbidity (%)	Presence	42.8	37.2	30.0	
	Absence	57.2	62.8	70.0	X^2^ = 24.4; *P* <0.05
Type of comorbidities (%)	Arterial hypertension (AHT)	28.4	33.2	33.0	
	Type 2 diabetes (T2D)	17.9	17.9	18.7	
	Dyslipidemia (DL)	10.1	2.6	9.8	
	Obesity	10.1	2.6	1.3	
	Mental disorder (MD)	2.3	12.7	2.4	
	Hypothyroidism (HT)	4.5	2.2	4.7	
	Acute myocardial infarction (AMI)	2.3	2.6	7.1	
	Pulmonary fibrosis (PF)	0.3	0.4	11.1	
	Asthma (AS)	4.6	3,5	1.6	
	Insulin resistance (RI)	3.0	5.2	1.1	
	Chronic kidney disease (CKD)	3.3	0.4	2.9	
	Chronic obstructive pulmonary disease (COPD)	2.1	1.3	0.8	
	Heart failure (HF)	1.5	2.6	1.1	
	Epilepsy (EP)	1.0	4.4	0.8	
	Dementia (DM)	2.1	0.4	0.5	
	Cardiovascular accident (CA)	1.7	0.9	1.1	
	Valvulopathy (VA)	0.5	0.9	1.3	
	Rheumatoid arthritis (RA)	0.8	1.7	0.0	
	Atrial fibrillation (AF)	1.2	0.4	0.0	
	Inflammatory bowel disease (ABD)	0.3	2.6	0.0	
	Fibromyalgia (FB)	0.5	0.4	0.3	
	Tuberculosis (TBC)	0.5	0.0	0.0	
	Chronic liver damage (CLD)	0.3	0.0	0.3	
	Down's Syndrome (DS)	0.2	0.9	0.0	
	Celiac Disease (CD)	0.3	0.0	0.0	
	Myasthenia gravis (MG)	0.2	0.0	0.0	
	Parkinson's	0.0	0.0	0.3	X^2^ = 136.4; *P* <0.05

**Table 2:** Prevalence of symptomatology by patients in the three periods analyzed.

**Symptoms**	**1^**st**^ wave (%)**	**Inter-wave (%)**	**2^**nd**^ wave (%)**
Cough	16	14	20
Headache	14	13	19
Myalgias	14	13	8
Fever	10	5	9
Anosmia	8	10	13
Respiratory Distress	8	11	13
Odynophagia	8	7	11
Gastrointestinal disease	7	9	14
Ageusia	7	9	11
Others	5	7	8
Asthenia	3	2	1

The mortality rate during the first wave was 4.5, 1.5 in the inter-wave, and 0.6% in the second wave (Table 1), decreasing significantly between the first and second wave (*X*^2^ = 24.6; *p* < 0.05). The lower mortality in the second wave could be associated with a lower viral load among infected patients, where in the first wave of infection it was 1.15 × 10^7^ ± 5.37 × 10^7^, inter-wave; 1.73 × 10^6^ ± 7.0 × 10^6^, and the second wave of infection it was 4.56 × 10^5^ ± 2.64 × 10^5^ viral copies/μl (Table 1).

On the other hand, male patients show a higher level of severity (ICU admission) than women (*F* = 5.2, *p* < 0.05). By the end of the analyzed periods, the total number of deaths was 39 patients (22 men and 17 women) (Table 3), registering no significant differences between sexes (*X*^2^ = 1.5; *p* < 0.05) in the periods analyzed. The prevalence of infections in women and men does not have significant differences, showing the same susceptibility to COVID-19 infection in the analyzed data (Supplementary Table S1). Based on these analyses, we determined a decrease in the age of the infected patient, a reduction in the incidence of severe illness, and less duration of the symptoms at the second wave.

**Table 3:** Deceased and recovered by gender, in the three periods analyzed (number).

	**Men**	**Woman**	**Total**
Recovery	769	886	1,655
Deceased	22	17	39
Total	791	903	1,694

*X^2^ = 1.5; P < 0.05*.

### Comorbidities in COVID-19 Severity

From the 1,694 positive-diagnosed patients, the number of comorbidities decreased in the second wave of infections. Thus, we registered 42.8% for the first wave, while the number of comorbidities decreased to 30% for the second wave (Table 1, *X*^2^ = 136.4, *p* < 0.05). The most common of them were arterial hypertension (AHT) and type 2 diabetes (DM2) for the three periods. The other comorbidities were about 10% or less frequent. Patients with comorbidities registered a close association with serious illness (*F* = 70.4, *p* < 0.05), showing an average of 2.3 comorbidities in deceased patients compared with recovered patients (0.7) (*F* = 70.4; *p* < 0.05). Severity was also related to the gender of each patient, since men showed more admission to ICU/ITU (*F* = 5.2, *p* < 0.05). Furthermore, there is no relationship between the type of comorbidity and the survival rate of the patients (Table 4). Therefore, these data suggest that increasing the number of comorbidities in a patient increases the risk of generating severe COVID-19. At the same time, the data are not sufficient to relate the specific types of comorbidities to the severity of the disease.

**Table 4:** Main types of comorbidities and their relationship with recovery or death from COVID-19 disease (*n*).

	**AHT**	**T2D**	**DL**	**Obesity**	**MD**	**HT**	**Others**	**Total**
Recovery	346.9	199.1	99.0	67.0	52.0	47.0	255.2	1,066
Deceased	26.1	20.9	5.0	6.0	0.0	3.0	21.8	83
Total	373	220	104	73	52	50	277	1,149

*X^2^ = 7.1; P > 0.05. AHT, Arterial hypertension. T2D, Type 2 diabetes. DL, Dyslipidemia. MD, Mental disorder. HT, Hypothyroidism*.

### Vaccination Effect in the Second Wave

**Figure 3:**
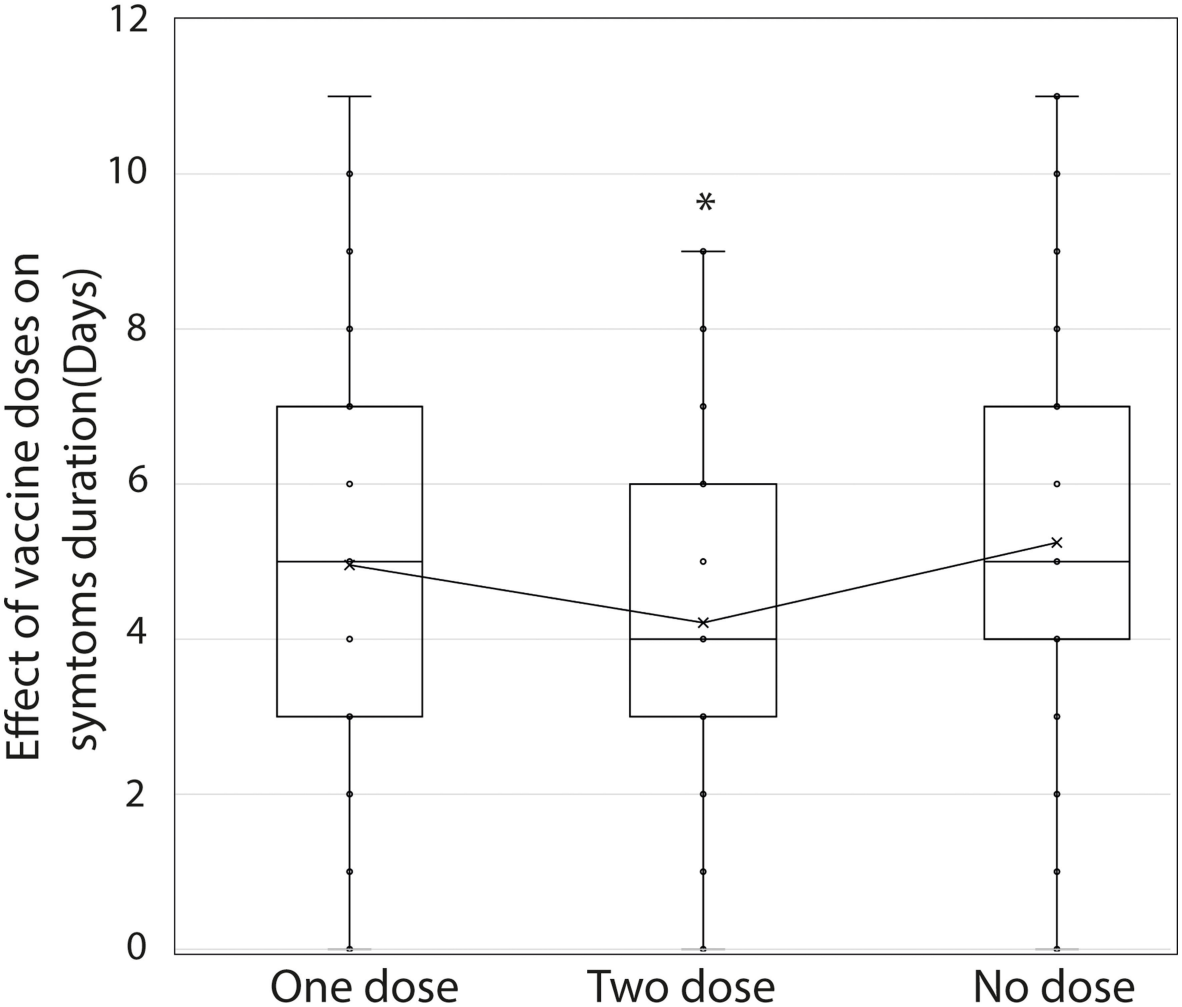
Effect of vaccination on the duration of COVID-19 symptoms. The period in days of duration of symptoms in patients with incomplete vaccination (one dose), complete vaccination (two doses), and without vaccination (no dose) during the second wave is shown. *p < 0.05. Analysis performed with a chi-square test.

In Chile, the vaccination process began in December 2020. The patients from the second wave showed less severity, suggesting that the less severe outcome is associated with the decrease in the age of the patients but not with the vaccination, since only people older than 60 years had been vaccinated at that time. The second wave consisted of 705 patients, of whom 451 were not vaccinated, 141 received both doses (full vaccinated), and 113 had only one dose (incomplete vaccination schedule). The distribution of hospitalized patients was 12.4% for those with an incomplete vaccination schedule, 7.1% for patients with two doses, and 7.1% for unvaccinated patients, concerning the total number of patients in each group analyzed. Of the total hospitalized, 25, 17.86, and 57.14% were patients with incomplete, complete, and no vaccine schedules, respectively. However, most of the non-hospitalized patients had no vaccine schedule (64.56% of the total cases) (Table 5). These differences were not significant, indicating that more data are needed to determine the actual contribution of vaccination to hospitalization and severe COVID-19. Importantly, patients with a complete vaccination scheme (two doses) presented a reduction in the duration of symptoms compared with incompletely vaccinated (one dose) and unvaccinated (no dose) patients (Figure 3, *F* = 12.0, *p* < 0.05). Unexpectedly in the second wave, even though complete vaccination in patients showed a decrease in the severity of COVID-19, it was related to a higher viral load than patients with incomplete or no vaccination schedule (*F* = 6.8, *p* < 0.05). These results suggest that the vaccination process only reduces the duration of symptoms in infected patients. Importantly, in Chile the people 60-years older were the first group to be vaccinated with Pfizer and CoronaVacVaccine until June 2021. Thus, we hypothesize the severity of COVID-19 in the youngest population could be related to a delay in the administration of the vaccine.

**Table 5:** Relationship between vaccination schedule (incomplete, full, or unvaccinated) and hospitalization of patients infected with COVID-19 in the second wave.

	**Hospitalization**		**No hospitalization**		
	**% from subgroup patients**	**% subgroup in total hospitalized patients**	**% from subgroup patients**	**% subgroup in total no. hospitalized patients**	**Total Patients (*n*°)**
Incomplete vaccination schedule	14/113 (12.40%)	14/56 (25.00%)	99/113 (87.6%)	99/649 (15.25%)	113
Full vaccination schedule	10/141 (7.10%)	10/56 (17.86%)	131/141 (92.9%)	131/649 (20.18%)	141
No vaccinated	32/451 (7.10%)	32/56 (57.14%)	419/451 (92.9%)	419/649 (64.56%)	451
Total	56		649		705

*X^2^ = 3.6; P > 0.05*.

## Discussion

Previous retrospective studies have documented the behavior of SARS-CoV-2 infection waves in different localities, countries, and patient groups. For example, Coyer et al. (12), reported a higher infection rate in minority ethnic groups than those of Dutch origin during the second wave in Amsterdam (12). On the other hand, Vinceti et al. (13) indicated that Italian provinces with a higher incidence of infections in the first wave experienced lower incidences in the second, possibly due to the early generation of local immunity (13), which, according to a study in China, can last up to 9 months (14). In contrast, a study in India indicated a sharp increase in the spread of SARS-CoV-2 during the second wave compared with the first wave apparently due to the appearance of new and more contagious variants, thus generating a higher mortality rate, especially in older patients associated with comorbidities (15). Another study reported younger patients infected in the second wave in Reus (Spain), although with shorter hospitalizations and a lower mortality rate than in the first wave (7). In addition, a more recent study indicated that the fourth wave of infections generated by the Omicron variant was less lethal than that previously generated by the third wave associated with the Delta variant in South Africa (16). These antecedents indicate that there are different behaviors of the waves of contagion by SARS-CoV-2 in different parts of the world. In our study, we compared for the first time the behavior of patients infected with SARS CoV-2 between the first two pandemic waves in a Chilean primary health center. We found a decrease in mortality related to COVID-19 during the second wave, with an age shift toward younger patients. At the same time, COVID-19 severity was associated with a greater number of comorbidities in infected patients. Although the unvaccinated group shows the highest percentage of hospitalized and non-hospitalized patients, our data showed that vaccination was not related to avoiding serious cases of COVID-19 in the second wave. Our results are similar to those published by Minnai et al. (9), who reported a lower mortality rate after the second wave, while the younger cases were associated with a lower rate of disease severity, although this severity was increased in the masculine gender (9), as was observed in our study. In a similar way to the behavior of the first and second waves in Chile, Iftimie et al. (7) reported that the second wave was recorded in younger people, while comorbidities were a determining factor in generating a severe COVID-19 disease in Spain (7). Therefore, these antecedents suggest that the virus behaved similarly in Chile, South America, the Mediterranean area, and Western Europe. However, although Chile presented similar behavior in the first wave of the pandemic compared with other countries (such as, Spain and Italy), we observed that a lower mortality rate occurred in the first wave compared with other European countries (17, 18). This does not necessarily indicate better public policies of the authorities since factors, such as geographic location, the genotype of the patients, the capacity of the health system, and population behavior itself are also variables for controlling the disease. Certain limitations of our study are worth noting. First, our data only represents a primary health center in Santiago of Chile. However, the data shown here can give an idea of the behavior of the pandemic in a specific population. Second, in the second wave, no difference was made between types of vaccine manufacturer nor in the days elapsed since the doses were administered after infection by COVID-19. Third, although it has been reported that vaccination significantly reduces the severity of COVID-19 (19), our data did not reduce the severity of COVID-19 positive cases, according to hospitalization criteria. Finally, the relationship between comorbidities and age cannot be established since older people have a greater number of comorbidities, which, in effect, are the focus of study in a SARS-CoV-2 infection (20).

On the other hand, circulating variants of SARS-CoV-2 were not considered in this report. It is interesting to note that in the second wave period, the predominant variants in Chile were Gamma and Alpha (21), which have been related to greater viral spread (22) and they are responsible to cause second waves of infections in other countries (23). These variants have been also associated with higher mortality, longer duration of symptoms, and viral load in the second wave. Importantly, this effect was not reflected in our data.

Collectively, this is the first retrospective report on the behavior of the first and second waves of the pandemic in a population of Chile. However, it must be considered that this is reflected by data from only one public health center that has been uninterruptedly monitored during the pandemic in Santiago de Chile. This study reports valuable information on the COVID-19 behavior and the result of the strategies applied during the pandemic.

## Conclusion

Our analyses indicate a lower rate of deaths associated with COVID-19 in the second wave than in the first wave in a public primary health center in Santiago de Chile. According to our statistics, the group of patients infected in the second wave was younger than in the first wave. This antecedent is related to a lower number of associated comorbidities, a critical factor in the risk of severe COVID-19 disease. Finally, vaccination was effective in reducing the duration of symptoms and viral load.

## Data Availability Statement

The raw data supporting the conclusions of this article will be made available by the authors, without undue reservation.

## Ethics Statement

The studies involving human participants were reviewed and approved by the Ethical Committee of the University of Santiago of Chile (No. 226/2021) and the Scientific Ethical Committee of the Central Metropolitan Health Service, Ministry of Health, Government of Chile (No. 370/2021). Written informed consent for participation was not required for this study in accordance with the national legislation and the institutional requirements.

## Author Contributions

Conceptualization and data curation: CA-C and FER-L. Methodology: CA-C and EV-V. Validation: FER-L, AMS, and MI. Formal analysis: SC. Investigation: AM-T, RL, AI-M, AE, SR-C, DT-A, SM-C, and CT. Resources: CA-C, MI, FER-L, and AMS. Writing—original draft preparation: CA-C. Writing—review and editing: FER-L and CB-A. Visualization: DR and EL-S. Supervision: CA-C, KM, DV, FER-L, and AMS. Project administration and funding acquisition: AMS and FER-L. All authors contributed to the article and approved the submitted version.

## Funding

The Laboratory of Virology had the support from the COVID-19 diagnosis in the University laboratories network (Ministry of Sciences, Ministry of Health, Government of Chile) for diagnosis tasks. The authors also thank the Rapid Assignment of Resources for Research Projects on the Coronavirus (COVID-19) (Project Number COVID1038; AMS, ANID, Government of Chile), Fondecyt regular project numbers 1201664 (MI) and 1211841 (FER-L) (ANID, Government of Chile), Fondecyt iniciación 11221308 (EV-V) (ANID, Government of Chile), and DICYT-USACH Project Number 021943AC (CA-C.) grants. EV-V was partially funded by FONDEQUIP grant project number EQM200016, and Basal project CEDENNA AFB-180001 (ANID, Government of Chile). The funders had no role in the study design, data collection and analysis, decision to publish, or preparation of the manuscript.

## Conflict of Interest

The authors declare that the research was conducted in the absence of any commercial or financial relationships that could be construed as a potential conflict of interest.

## Publisher's Note

All claims expressed in this article are solely those of the authors and do not necessarily represent those of their affiliated organizations, or those of the publisher, the editors and the reviewers. Any product that may be evaluated in this article, or claim that may be made by its manufacturer, is not guaranteed or endorsed by the publisher.
