# Supplementary material for: Comparison of the First and Second Wave of Infections by SARS-CoV-2: A Retrospective and Longitudinal Study From a Primary Health Care Center in Santiago of Chile
Source: Front Public Health. 2022 Jun 30;10:913519. doi: 10.3389/fpubh.2022.913519 (PMC9280347; doi:10.3389/fpubh.2022.913519)
Supplement: Supplementary file 1 [file Table_1.docx]

Supplementary Material

|  | Women | Men |
| --- | --- | --- |
| 1^st^ Wave | 49.7% | 50.3% |
| Inter-Wave | 49.4% | 50.6% |
| 2^nd^ wave | 50.4 | 49.6% |
| p<0.05 |  |  |

Supplementary Table 1. Prevalence of infections by gender from total positive COVID-19 tests
